# Decision-making psychological state and characteristic of proxies of thrombolytic patients: a pilot study

**DOI:** 10.1038/s41598-022-14124-x

**Published:** 2022-06-20

**Authors:** Guo Yuanli, Liu Yanjin, Guo Lina, Dong Xiaofang, Yang Caixia, Wang Min, Gao Huanhuan, Lv Peihua, Ma Keke

**Affiliations:** The First Affiliated Hospital of Zhengzhou University, Zhengzhou, China

**Keywords:** Neurological disorders, Human behaviour

## Abstract

Intravenous thrombolysis is the preferred treatment modality for acute ischemic stroke. In China, written informed consent from patients or proxies must be obtained before intravenous thrombolysis is performed, which always leads to in-hospital delay of thrombolysis. To explore the relationship between characteristics of thrombolysis decision-making and psychological states of proxies of AIS patients. This was a pilot study. 231 proxies of AIS patients were recruited, including 147 males and 84 females. STAI, WFPTS, CAOT, CPS, C-DCS and time-consuming of decision-making were collected by trained nurses during the period from signing informed consent to thrombolysis finished. The general information was collected within 24 h after admission. Pearson correlation analysis and the Ridge regression analysis were used to explore the correlation and causality between psychological indicators (STAI, WFPTS, CAOT, CPS) and decision-making characteristics (C-DCS, Time consuming). Structural equation modeling was used to explore the direct and indirect effect of psychological factors on decision-making characteristics. The mean of anxiety, trust in physicians, and decision conflict were 49.20 ± 9.50, 37.83 ± 6.63 and 30.60 ± 14.77, respectively. The CAOT was associated with C-DCS through the mediation of STAI and WFPTS (*p* < 0.001). The CAOT was associated with time-consuming through the mediation of STAI, WFPTS and CPS (*p* < 0.05). The CAOT, STAI and WFPTS were associated with C-DCS (*p* < 0.05), and STAI, WFPTS and CPS were associated with time-consuming (*p* < 0.01). The proxy of patients with acute ischemic stroke had severe decision conflict in thrombolysis decision-making. The psychological state was associated with decision conflict and the time-consuming. Medical staff should explore methods to release the anxiety and increase the trust in physicians to reduce the decision-making conflict and time-consuming, which could promote the smooth progress of the informed consent.

## Introduction

The overall lifetime risk of stroke in China is 39.9%, ranking first in the world^1^, and acute ischemic stroke (AIS) accounts for 80% of all stroke types^2^. Intravenous thrombolytic therapy with tissue-type plasminogen activator (tPA) is the preferred treatment for AIS, but it is limited by a strict time window, with 1.9 million neurons and 14 billion synapses dying every 1 min of delay^3^. If thrombolysis was delayed, patients have few opportunities to regain an ideal functional recovery and the risk of bleeding after thrombolysis is elevated^4^. Thus, the completion of reperfusion therapy in the shortest time is a prerequisite for optimal clinical outcomes. However, only 33.4% of patients received tPA within 4.5 h of stroke in China, according to the data from the National Stroke Registry Center^5^. Many researchers have reported the factors of thrombolysis delay, including prehospital factors and in-hospital factors, such as insufficient recognition of stroke symptoms^6^, transport methods^7^, knowledge about AIS^8^, and education degree of the decision-makers^9^. One of the strategies to deal with the barriers was to improve the process of informed consent for thrombolysis^10^.

Unlike other countries with relatively loose informed consent policies, it’s mandatory and must be in written form to obtain the informed consent before the initiation of thrombolysis in China at present. So it is not yet possible to reduce time delayed by canceling informed consent as guideline^2^ recommended or changing it into oral form as Singapore researcher reported^11^. The decision-making process of thrombolytic therapy made the decision-makers face the dual pressure of closure time and the perceived risk of hemorrhage, which requires a large amount of information acquisition and processing, value judgment and trade-off in a limited time, especially in China, where the written informed consent was mandatory. Thus, we need to dissect the process of informed consent from a different perspective in China, rather than a simplification of the procedure. For example, we should probably regard the decision-making process of proxy as a psychological one and explore the barriers to rapid access to informed consent from a psychological perspective. In this study, we would explore the relationship between the psychological state and decision-making characteristics of proxy.

The thrombolysis decision-making is essentially a process of risky decisions under time pressure. Some researchers have confirmed the relationship between emotions and risk decision-making outcomes such as anxiety, but there were inconclusive results on whether facilitative or inhibitory effects^12,13^. The interactive effects of time and emotion also play an important role in the decision-making process^14^. However, since the aspiration of a shorter time of DNT (Door to Needle Time), we have been focusing solely on the time spent on the informed consent, neglecting how decision-makers felt in the decision-making process and whether it caused prolonged decision-making. Researchers had reported that 1.2–98% of thrombolysis decision was made by proxies^15,16^. Guo found that most thrombolysis decisions were made by proxy decision makers, and patients with AIS and proxy decision makers did not feel and think exactly the same in the decision-making process in a qualitative study^17^. Thus, we would investigate proxy decision makers' anxiety levels, cognitive dispositions, and trust in physicians, as well as their relationship to the decision-making, including the level of decision conflict and time consuming on decision making in this study.

## Method

### Design and sample

This was an observational study from an advanced stroke center in China. This study aimed to explore the psychological state and decision-making characteristics of proxy decision makers, and the relationship between them in the thrombolysis decision-making process. The results of our previous qualitative research^17^ showed that patients who made decisions by themselves also had decisional conflicts, but their considerations during decision-making process were different from those of their proxies. For example, patients consider more about whether they would bring a serious economic burden to their families, while their families thought more about problems from the perspective of patients' prognosis, so we were worried that these differences will affect the analysis of several variables. Thus, self-deciding patients were excluded in this study. Patients were recruited from the emergency department from 1st September 2020 to 30th September 2021. Patients were eligible if they were diagnosed with acute stroke according to the Chinese guidelines for the intravascular treatment of acute ischemic stroke 2018^18^. Inclusion criteria: (1) patients met the sign for thrombolysis and signed written informed consent for thrombolysis by proxy. (2) be able to understand and express the Chinese language. Exclusion criteria: (1) Thrombolysis decisions made by patients themselves. The study was approved by the Ethics Review Committee of life sciences in the First Affiliated Hospital of Zhengzhou University (2020-KS-HNSR071).

### Measurements

#### Cognitive appraisal orientation

Cognitive appraisal orientation test (CAOT) was developed by Scheier^19^ in 1994, it comprises 10 items, including 3 items of pessimism which assess an individual's tendency to predict how things develop negatively, 4 items of optimism which assess an individual's tendency to predict how things develop positively and the 3 other items. The 5-point Likert scale was used (0–4 points). A higher total score indicates a more positive cognitive disposition. In this study, the Chinese version by Yong Zhang^20^ in 2006 was used. The Cronbach’s α for internal consistency was 0.833 in this study.

#### State anxiety

The state/trait inventory (STAI) was compiled by Charles Spielberger in 1977 and revised in 1983^21^. It is a self-evaluation questionnaire, which can directly reflect the subjective feelings of anxiety and distinguish current (state anxiety) from consistent (trait anxiety). In this study, we aimed to explore the anxiety state of the gents in the decision-making process, so we only used the former 20 items to assess the current anxiety of proxy. 4 grades scoring method (1–4, completely not ~ severe) was used to calculate the score. Items of positive emotions were reverse scored. Higher scores showed more severe anxiety. The Cronbach’s α for internal consistency was 0.879 in this study.

#### Proxies’ trust in their physicians

The Chinese version of the Wake Forest Physician Trust Scale (WFPTS) was revised by Dong^22^ in 2012. The revised scale includes 10 items of 2 dimensions named “charity” and “professional skills”. The “charity” means physicians’ concern and attitude toward patients and proxy and the ability to communicate with them. The “professional skills” means diagnostic and therapeutic capabilities. Each item was scored from 1 (very disagree) to 5 (very agree). Higher total scores mean more trust in physicians. The Cronbach’s α for internal consistency was 0.704 in this study.

#### Decision conflict

The Chinese version of the Decision Conflict Scale(C-DCS) by Yu^23^ was used to evaluate the certainty of agents’ choice of thrombolytic therapy. The scale includes 16 items in 5 dimensions, including information, values, decision support, decision effectiveness and decision certainty. The Likert 5-level scoring method was adopted, and we assign 0–4 points from ‘yes’ to ‘no’. The total score after transformation ranges from 0 to 100 points, where < 25 points are effective in decision-making, 25–37.4 points are conflict in decision-making, and ≥ 37.5 points are delay in decision-making or uncertainty in decision-making. The Cronbach’s α for internal consistency was 0.968 in this study.

#### The control preference scale

The control preference scale (CPS), a card sort technique with five options, was developed by Degner^24^. The Chinese version was translated and tested by Xu^25^ to ask patients how they expect to be involved in choosing treatment options. The cards ranged from a more passive role (cards A and B), through a collaborative role (card C) to a more active role (cards D and E). Subjects were required to choose the most suitable decision-making role among the five decision-making roles according to the detailed description. The Cronbach’s α for internal consistency was 0.873 in this study.

#### Time consuming for decision making

We defined the period from the initiation of the informed consent talk to the signing off by proxy as time consuming (TC) for decision making.

#### General information

The general information questionnaire was developed by researchers according to the aim of this study, which included sociodemographic data (age, sex, education of proxy, relationship with patients, financial burden) and disease related data (comorbidities, recurrence, NIHSS before thrombolysis).

The Cronbach’s α of the total scale in this study was 0.831.

### Data collection

The STAI, C-DCS, WFPTS, CPS and the CAOT were filled in sequentially under the guidance of nurses from signing informed consent to the end of thrombolysis. The time from consent talk began to sign informed consent was calculated and recorded by nurses. The general information was collected by nurses within 24 h of admission. All the data were collected in an electronic questionnaire by trained nurses, who were not the same as the nurses who conducted data analysis.

### Sample size

There were 10 variables were analyzed in this study. We calculated the sample size based on the principle of five to ten times the number of variables. The smallest sample size was 50.

### Data analysis

Normality test was conducted to explore the distribution of the data. Normally distributed data was described using mean and standard deviation, and skewed distributed data were reported using median, first quartile and third quartile. Categorical variables were described using percentages. The Pearson correlation analysis was conducted to explore the relationship between the psychological states (CAOT, STAI, CPS, WFPTS) and decision-making characteristics (C-DCS and TC). Variance Inflation Factor (VIF) diagnosis was conducted to analyze the collinearity of independent variables. When the collinearity of the independent variable is acceptable (VIF < 10), multiple linear regression was conducted to explore the effect of the psychological states on decision-making characteristics, or the Ridge regression analysis would be used. The structural equation model (SEM) was used to verify the path. A two-tailed significance level of 0.05 was used. All the data analysis was conducted using the SPSSAU 21.0, which is an online application software retrieved from https://www.spssau.com.

### Ethical considerations and informed consent statement

The study was conducted according to the guidelines of the Declaration of Helsinki. All the participants were previously informed of the detail of the research and signed the informed consent documents. The subjects' personal information could not be viewed by anyone outside the study group. The study was approved by the Ethics Review Committee of life sciences in the First Affiliated Hospital of Zhengzhou University (2020-KS-HNSR071).

### Ethics approval and consent to participate

This study has the approval of the Ethics Review Committee of life sciences in the First Affiliated Hospital of Zhengzhou University (2020-KS-HNSR071) and informed consent was obtained before all questionnaires were collected.

## Results

231 proxies of AIS patients were recruited in this study, including 147 males and 84 females. The median of NIHSS was 4(2.6). The age of patients and proxies were 59.61 ± 13.03 and 41.95 ± 9.98, respectively. Family roles of proxies comprised 59.8% sons or daughters, 19.5% spouses, 10.4% parents, 5.2% brothers or sisters, and 5.2% others. Over half of the proxy had a bachelor's degree and above. Some other information was shown in Table 1.

**Table 1:** General information of subjects (n = 231).

Index	Frequency (percentage)	Index	Frequency (percentage)
***Proxy sex***		***Number of recurrences***	
Male	147(63.6)	0	147(63.6)
Female	84(36.4)	1	69(29.9)
***Family role of proxy***		2	12(5.2)
Sons or daughters	138(59.8)	3	3(1.3)
Spouse	45(19.5)	***Kinds of comorbidity***	
Parents	24(10.4)	0	45(19.5)
Brother or sister	12(5.2)	1	114(49.4)
Others	12(5.2)	2	48(20.8)
***Economic burden caused by patients' illness***		3	21(9.1)
None	63(27.3)	4	3(1.3)
Mild	99(42.9)	***Education level of proxy***	
Middle	45(19.5)	Primary and below	3(1.3)
Large	24(10.4)	Junior middle school	48(20.8)
***Patient sex***		Senior high school	54(23.4)
Male	168(72.7)	Bachelor	105(45.5)
Female	63(27.3)	Master	21(9.1)

The average score of state anxiety of proxies was 49.20, which was higher than that of normal people (39.71 ± 8.89, 38.97 ± 8.45) in China^26^. The average score of C-DCS was 30.60. 54.8% of proxies scored ≥ 25, which meant decision conflict existed. 22.1% of proxies scored ≥ 37.5, which meant probable decision delay and uncertainty. The time-consuming of thrombolysis was between 1.5 and 38 min, with a median of 9 (6, 11) minutes. 17 proxies spent over 15 min on decision-making. The results of CPS showed that 40.26% proxies were passive decision-makers and 42.86% were collaborative ones. The scores of trust in physicians, cognitive appraisal orientation and the 5 dimensions of C-DCS were shown in Table 2.

**Table 2:** Psychological indicators of proxies (n = 231).

Indicators	Mean value ± standard deviation
STAI	49.20 ± 9.50
WFPTS	37.83 ± 6.63
CAOT	37.30 ± 6.07
C-DCS (information)	6.30 ± 3.22
C-DCS (value)	6.08 ± 3.30
C-DCS (support)	5.86 ± 3.17
C-DCS (certainty)	5.65 ± 2.93
C-DCS (effective)	6.71 ± 4.04
C-DCS(Total)	30.60 ± 14.77

The correlations between STAI, WFPTS, CAOT, CPS, C-DCS and Time consuming were shown in Table 3. The results showed STAI had a positive correlation with C-DCS and Time consuming, and WFPTS had a negative correlation with C-DCS. CPS was positively correlated with Time consuming.

**Table 3:** Correlation between STAI, WFPTS, CAOT, CPS, C-DCS and Time consuming (n = 231).

		C-DCS	Time consuming
STAI	Correlation coefficient	0.284	0.207
	*p*	< 0.001	0.002
WFPTS	Correlation coefficient	− 0.677	− 0.034
	*p*	< 0.001	0.602
CAOT	Correlation coefficient	0.104	− 0.049
	*p*	0.113	0.459
CPS	Correlation coefficient	− 0.076	0.206
	*p*	0.247	0.002

**Figure 1:**
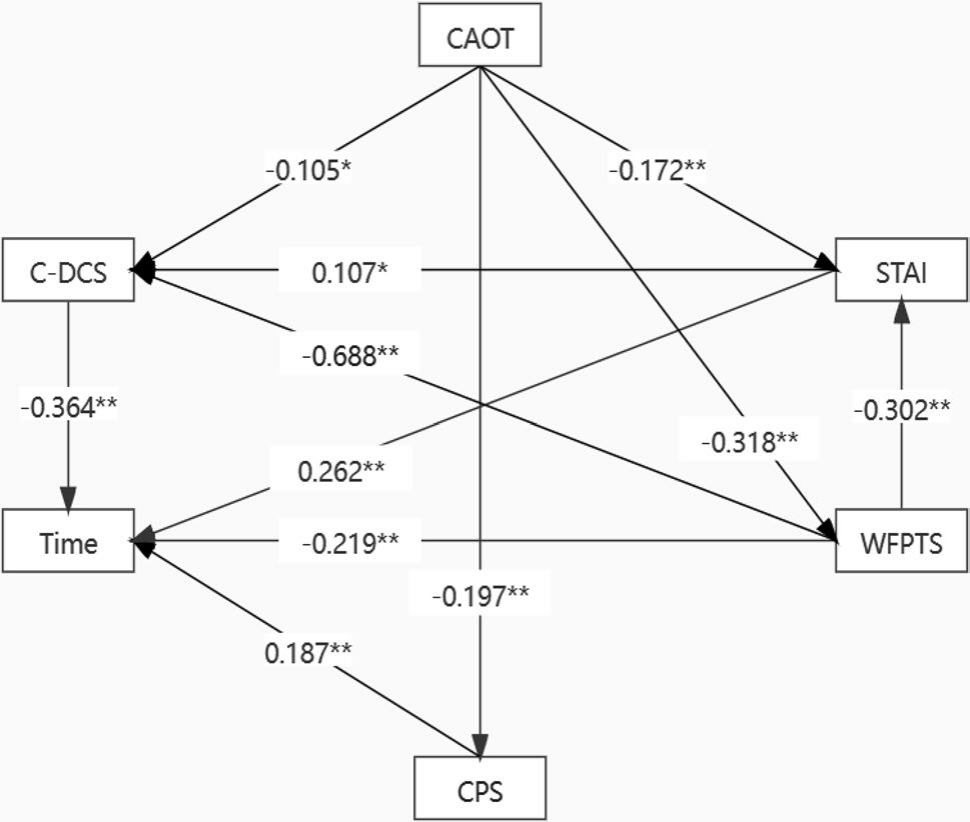
The pathway of SEM.

Because of the serious collinearity of independent variables (Variance Inflation Factor > 10), Ridge regression analysis was used to explore the influence of psychological states on decision-making characteristics. The K value was set as 0.3 when the dependent variable was C-DCS and 0.35 when the dependent variable was Time consuming according to the Ridge Trace. The results of Ridge regression analysis results showed that the psychological state was associated with decision-making characteristics, as shown in Tables 4 and 5. The results of pathway analysis showed that the CAOT was associated with the C-DCS and time consuming, and STAI, WFPTS and CPS were mediator between them. The STAI and the WFPTS were associated with C-DCS and time-consuming, and the STAI was the mediator between the WFPTS and time-consuming. The CPS was associated with the time-consuming, see Table 6. The goodness-of-fit indices were calculated to evaluate the model, as detailed in Table 7, which indicated that the model was accepted and significant. The pathway of effect was shown in Fig. 1 and the R^2^ of dependent variables were shown in Table 8.

**Table 4:** Ridge regression analysis results (Y: C-DCS).

Variables	Unstandardized Coefficients		Standardized Coefficient	t	*p*	R^2^	Adjusted R^2^	F
	B	SE	Beta					
Constant	72.851	7.675	–	9.492	< 0.001**	0.517	0.486	F (14,216) = 16.516, *p* < 0.001
Sex of proxy	2.648	1.135	0.086	2.333	0.021*			
Age of proxy	− 0.126	0.056	− 0.085	− 2.250	0.025*			
Relationship with patient	0.314	0.236	0.050	1.332	0.184			
Education of proxy	− 1.72	0.549	− 0.118	− 3.133	0.002**			
Financial burden	− 0.279	0.597	− 0.018	− 0.468	0.640			
Sex of patients	1.389	1.221	0.042	1.138	0.256			
Age of patients	0.056	0.042	0.050	1.333	0.184			
Complications	0.057	0.599	0.004	0.095	0.924			
NIHSS	0.252	0.102	0.091	2.459	0.015*			
Recurrence	0.313	0.268	0.044	1.170	0.243			
STAI	0.162	0.058	0.104	2.798	0.006**			
WFPTS	− 1.129	0.083	− 0.507	− 13.615	< 0.001**			
CAOT	− 0.106	0.091	− 0.044	− 1.170	0.243			
CPS	− 0.899	0.548	− 0.061	− 1.641	0.102			

*p < 0.05 **p < 0.01. Dummy variables were set for categorical variables. Sex: 1 = male, 2 = female; Education degree: 1 = primary and below, 2 = junior middle school, 3 = senior high school, 4 = bachelor, 5 = master; Relationship with patient: 1 = sons or daughters, 2 = spouse, 3 = parents, 4 = brother or sister, 5 = others; Economic burden: 1 = none, 2 = mild, 3 = middle, 4 = large.

**Table 5:** Ridge regression analysis results (Y: Time consuming).

Variables	Unstandardized coefficients		Standardize coefficient	t	*p*	R^2^	Adjusted R^2^	F
	B	SE	Beta					
Constant	− 2.537	3.666	–	− 0.692	0.490	0.132	0.076	F (14,216) = 2.354, *p* = 0.005
Sex of proxy	− 0.155	0.546	− 0.013	− 0.284	0.777			
Age of proxy	0.016	0.027	0.029	0.614	0.54			
Relationship with patient	0.116	0.112	0.049	1.033	0.303			
Education of proxy	0.729	0.262	0.133	2.782	0.006**			
Financial burden	0.073	0.285	0.012	0.256	0.798			
Sex of patients	0.120	0.587	0.010	0.204	0.838			
Age of patients	− 0.016	0.020	− 0.038	− 0.798	0.426			
Complications	− 0.325	0.288	− 0.053	− 1.127	0.261			
NIHSS	− 0.051	0.049	− 0.049	− 1.042	0.298			
Recurrence	− 0.100	0.129	− 0.037	− 0.774	0.440			
STAI	0.105	0.028	0.180	3.773	< 0.001**			
WFPTS	0.003	0.040	0.003	0.066	0.947			
CAOT	− 0.008	0.043	− 0.009	− 0.180	0.857			
CPS	0.813	0.263	0.147	3.091	0.002**			

*p < 0.05 **p < 0.01. Dummy variables were set for categorical variables. Sex: 1 = male, 2 = female; Education degree: 1 = primary and below, 2 = junior middle school, 3 = senior high school, 4 = bachelor, 5 = master; Relationship with patient: 1 = sons or daughters, 2 = spouse, 3 = parents, 4 = brother or sister, 5 = others; Economic burden: 1 = none, 2 = mild, 3 = middle, 4 = large.

**Table 6:** The correlation among psychological states and decision-making characteristics (n = 231).

Model pathway	Non standardized path coefficient	SE	z (CR value)	*p*	Standardized path coefficient
CAOT → STAI	− 0.270	0.104	− 2.601	0.009	− 0.172
WFPTS → STAI	− 0.434	0.095	− 4.561	< 0.001	− 0.302
CAOT → CPS	− 0.032	0.011	− 3.055	0.002	− 0.197
CAOT → WFPTS	− 0.347	0.068	− 5.094	< 0.001	− 0.318
STAI → TC	0.152	0.037	4.127	< 0.001	0.262
CPS → TC	1.031	0.336	3.070	0.002	0.187
WFPTS → TC	− 0.182	0.069	− 2.628	0.009	− 0.219
C-DCS → TC	− 0.137	0.032	− 4.333	< 0.001	− 0.364
STAI → C-DCS	0.165	0.076	2.168	0.030	0.107
CAOT → C-DCS	− 0.255	0.122	− 2.092	0.036	− 0.105
WFPTS → C-DCS	− 1.525	0.115	− 13.259	< 0.001	− 0.688

**Table 7:** The goodness-of-fit indices of the SEM (n = 231).

Index	Χ^2^/df	GFI	RMSEA	CFI	NFI	NNFI	TLI	AGFI	IFI	
Acceptable standard	< 3	> 0.9	< 0.10	> 0.9	> 0.9	> 0.9	> 0.9	> 0.9	> 0.9	
Value	1.405	0.991	0.042	0.993	0.978	0.974	0.974	0.95	0.993

**Table 8:** The SMC R^2^ of dependent variable (n = 231).

Variables	R^2^
STAI	0.088
CPS	0.039
WFPTS	0.101
Time consuming	0.149
C-DCS	0.488

## Discussion

Thrombolysis was a time-sensitive therapy for AIS. However, in the emergency setting, patients often have impaired consciousness and cannot directly express their values and wishes regarding their treatment^27^. The physicians would request the proxies to decide whether to receive the thrombolysis after quickly explaining its risks and benefits of it. Then the proxies were placed in a situation of time pressure, uncertain risk or benefit, complex pathogenesis and treatment mechanism, as well as information overload, which could cause emotional stress and then affect the decision quality^28^. It was reported that anxiety was one of the important emotional distress that led to decision conflict^29^. So how did the proxies feel in this emergency and risk decision process, and would their emotional state affect the decision characteristics were all unknown. In this study, we explored the relationship between psychological state and the decision characteristics to help medical staff better understand the psychological response and decision-making characteristics of proxies when they were faced with the thrombolysis decision.

The results showed the proxies were more anxious (49.20 ± 9.50) than the normal person in a previous study in China (39.71 ± 8.89, 38.97 ± 8.45)^26^. It was similar to the results of a previous qualitative study^30^ showing that anxiety could occur when patients with atrial fibrillation balanced the risks of bleeding and thrombosis. The level of trust in physicians (37.83 ± 6.63) was lower than that in Petrocchi’s research^31^ (39.07 ± 5.71, 39.11 ± 5.82). These poor emotional states might result from the particularity of the situation—time sensitivity, uncertainty, risk, and life-threaten. Physician–patient communication played an important role in the physician–patient trust^32^. However, physicians were eager to explain the thrombolysis as soon as possible, having little time to pay attention to the communication skills and emotional state of proxies, as well as that how much information was understood by listeners, which pushed decision-makers into a psychological conflict between lack of information and full of responsibility. All of these could lead proxies into a bad psychological state.

The quality of decision-making included a time-consuming of the process and proxies’ certainty about the decision^33^, which was assessed by C-DCS and decision time consuming in this study. The results showed that more than half of the proxies had decision conflict in the decision-making process. The dimension of effectiveness scored highest (6.71 ± 4.04) and then information (6.30 ± 3.22), which meant that in the decision dilemma, what bothered proxies most was that they were unsure whether the best decision for the patients had been made, and they were obliged to make the major decision when the relevant information was not fully acquired or understood. The results showed proxies spend 9 (6,11) minutes making thrombolysis decisions, meeting the standards (15 min) recommended in the Chinese guidelines^34^ but was higher than the medium time consuming (5 min) reported by Chai^35^. However, the longest time consuming was 38 min and there were still 17(7.36%) proxies spent more than 15 min on decision-making. Thus, we still need to make efforts to reduce the time-consuming on decision-making process except for the wide-used visual decision aids^36^. The results of this study showed that women faced more serious decisional conflict than men. The decision-making process was influenced by personal characteristics, such as sex and personality^37^. The research of Zhang revealed female showed higher neuroticism than male in risk decision-making, leading to pessimistic prediction of consequences and sensitivity to decision risks^37^. Thus, female tended to refuse thrombolysis to avoid hemorrhagic transformation risk in decision-making.

The results of SEM analysis showed that the STAI, WFPTS and CPS were mediators between CAOT and decision characteristic(*p* < 0.05), which means that an optimistic proxy would suffer from less anxiety which contributed to slighter decision conflict and less time consuming, which was in line with the findings of Beard^38^. According to the cognitive tendency questionnaire^20^, an optimistic person usually predicts that things will go in a good direction. Thus, an optimistic proxy might be not aware of the critical situation of AIS patients and perceived inadequate risk and harm of thrombolysis delay, which could not make them anxious and feel less difficult to make a decision. A special result in this study indicated that optimistic proxies showed less trust in the physician, and then resulted in more severe decision conflict and longer time consuming. It might be because that the severity of the disease and the emergency informed by the physicians contradicted the prediction of optimistic proxy that the event will develop in a good direction (the disease will turn for the better). As a result, they thought physicians overemphasized the danger of the disease only for the benefit of the hospital. However, the cognitive appraisal was unlikely to be changed in a few minutes in the emergency department before the thrombolysis started. Thus, we should not hope to ease their decision-making dilemma by reversing their cognitive appraisal. This result only reminded us that the prediction based on cognitive appraisal could affect the decision-making quality of proxies.

The results indicated that the proxies with more severe anxiety had higher decision conflicts and spent longer time on decision making. It was consistent with previous research indicating that anxiety could impair decision-making capacity^39^. It might be because that anxiety could weaken the allocation of cognitive resources, and then inhibit individual cognitive processing, resulting in the lack of cognitive resources and the inability to make correct judgments^40^. Besides, higher state anxiety was predicted more likely to avoid risk under time pressure^41^. As a result, the proxies had to make a hasty decision in a strict time-window which may against their true will at that moment, leaving the risk and benefit not fully understood, which made them feel like they were uncertain whether they had made an effective decision and fall into the decision conflict. Besides, we also found that anxiety mediated the correlation of trust and decision conflict as well as time consuming. Madrigal^42^ reported that in a state of high anxiety, increasing trust in physicians was associated with a lower preference for autonomous decision making, which was proved beneficial for accelerating the informed consent (Table 6: CPS → TC, *p* = 0.002, Standardized path coefficient = 0.187). This finding reminded us that medical staff should take measures to make the proxies calm and trust in physicians to understand and accept what they said and speed up the informed consent. For example, use communication skills in the process of informed consent or construct an artificial intelligence decision support system based on big data to increase the persuasiveness of physicians^43^.

We found that the higher CPS was associated with the longer time consuming, which meant that the proxies who hope to take part in the decision-making process as much as possible would spend more time making decision^25^. It was because the decision-making of thrombolysis must be based on sufficient information acquisition and understanding by decision-makers. Both of the two processes made proxies spent more time on information collection by all kinds of methods, including thinking by themselves and asking others except physicians. However, full shared decision-making was not well suited for the acute stroke scenario although it was highly valuable and appropriate in many medical decision-making situations, because of the lack of clinical equipoise regarding tPA administration, and the time-sensitive nature of this acute stroke treatment, and an inappropriate setting for in-depth discussions^44^. Thus, physicians should get the gist across in a more skilled way in informed consent to shorten the time consuming of proxies’ decision-making. Supplying simulation training for neurology residents on acquiring tPA consent^45^ might be helpful.

According to the results, the proxies faced with higher decision conflicts spent less time on making a decision, which seemed to puzzle but was reasonable when stood in the emergency environment of thrombolysis decision-making. According to the research of Keith E. Stanovich and Richard F.West^46^, there were two systems in the thinking process of decision, and system 1 was chosen unconsciously when the decision-makers faced with emergency, which was faster, more automatic and less dependent on computing capacity compared with the system 2 which was based on logical reasoning, and the two systems work alternately frequently. In the situation of thrombolysis decision-making, a proxy having more severe decision conflict probably made a decision based more on system 1, so they spent less time making a decision. However, it doesn’t mean that higher decision conflict should be expected, because higher decision conflict indicated more uncertainty and a negative experience for decision, which is not beneficial for establishing a good doctor-patient relationship or cause medical disputes once a complication occurs.

## The theoretical and practice implication

The results of this study provide a new perspective for the study related to thrombolytic delay in AIS patients in the future, which indicated that a psychological state is an important factor affecting the quality of decision-making. And the negative correlation between decision conflict and time consuming enlightened us that system 1 based on intuition in reasoning was more likely to be auto-started in the thrombolysis decision-making and further research should be conducted to explore the method improving decision-making quality based on the system 1. Besides, researchers and clinical physicians should pay more attention to the psychological state in emergency decision-making of thrombolysis and its impact on the decision results. Building trust in physicians of the public could be the first but difficult step to enhancing quickly informed consent in China. Taking quick and effective skills to reduce the anxiety of decision-makers may also come into effect.

## Conclusion

Proxies of AIS patients were anxiety in the thrombolysis decision-making and their trust in physicians was not optimistic. The Cognitive appraisal orientation (CAOT) was associated with decision-making characteristics (C-DCS and time consuming) based on the mediator of anxiety (STAI), trust in physicians (WFPTS) and the expectation of involvement in choosing treatment options (CPS). Higher anxiety during decision-making and less trust in physicians could cause more severe decision conflict of proxies and longer time consuming on decision-making. Proxies who are expected to take part in decision-making would spend more time consenting to thrombolysis.

## Limitation

In this study, patients who refused the thrombolysis and their proxies were excluded because the quality of decision-making assessment using D-CDS and time consuming was meaningful only if the patients received the thrombolysis. The “refusal” decision was regarded as a poor decision even if it spent a little time. However, it was equally important to learn about the psychological mechanism of refusing thrombolysis, which could help physicians persuade patients and proxies to consent. In a future study, qualitative research should be conducted to dissect the reason for their refusal. Besides, the sample in this study was recruited in a tertiary hospital, further researches need to be conducted to confirm the conclusion in other areas with different cultural background.

## Data Availability

The data relating to the current study are available from the corresponding author (M-KK) on reasonable request.
